# Long-term effect of transcranial direct current stimulation in the treatment of chronic tinnitus: A randomized, placebo-controlled trial

**DOI:** 10.3389/fpsyt.2022.969800

**Published:** 2022-10-13

**Authors:** Tadeas Mares, Jakub Albrecht, Jozef Buday, Gabriela Podgorna, Thai Hong Le, Eva Magyarova, Katerina Poshor, Jakub Halik, Jan Buna, Vaclav Capek, Lenka Kostylkova, Johana Klasova, Vratislav Fabian, Martin Anders

**Affiliations:** ^1^Department of Psychiatry, First Faculty of Medicine, Charles University and General University Hospital in Prague, Prague, Czechia; ^2^First Faculty of Medicine, Charles University, Prague, Czechia; ^3^Department of Psychiatry, Krajska zdravotni a.s. – Most Hospital, Most, Czechia; ^4^National Institute of Mental Health, Klecany, Czechia; ^5^Third Faculty of Medicine, Charles University, Prague, Czechia; ^6^Department of Internal Medicine, First Faculty of Medicine, Charles University and Military University Hospital, Prague, Czechia; ^7^Department of Physics, Faculty of Electrical Engineering, Czech Technical University in Prague, Prague, Czechia

**Keywords:** non-invasive, neurostimulation, tDCS, transcranial direct current stimulation (tDCS), protocol optimization, dorsolateral prefrontal cortex, tinnitus, bifrontal tDCS

## Abstract

**Introduction:**

Tinnitus is an intrusive and chronic illness affecting a significant portion of the population, decreasing affected individuals’ quality of life and socioeconomic functioning. Transcranial Direct Current Stimulation (tDCS) is a non-invasive neuromodulatory method utilizing weak electrical currents to elicit short and long-term central nervous system changes. Several studies have proven its effect on tinnitus. We aimed to broaden the knowledge and provide data on the effect and its retention.

**Methods:**

In the randomized, double-blinded, sham-controlled trial, 39 patients (active *n* = 19, sham *n* = 20) underwent bifrontal tDCS (anode over right dorsolateral prefrontal cortex (DLPFC), cathode left DLPFC, current of 1.5 mA, 20 min, 6 sessions in 2 weeks). Tinnitus Functional Index (TFI), Iowa Tinnitus Handicap Questionnaire (ITHQ), Beck Anxiety Inventory (BAI), Zung Self-Rating Depression Scale (SDS), and WHO-Quality of Life-BREF were employed in 4 evaluation points, including the follow-ups of 6 weeks and 6 months.

**Results:**

We reached a delayed, significant long-term improvement (*p* < 0.05) in auditory difficulties associated with tinnitus and noticed it even after 6 months compared to placebo. We also reached a short-term, negative effect in the psychological domain of WHO-Quality of Life-BREF (*p* < 0.05). Not all subdomains of TFI and ITHQ reached statistical significance during the data analysis, even though specific positive trends were noticed.

**Conclusion:**

We proved partial, positive, long-term effects of tDCS on tinnitus and short-term, negative, transient effect on a specific aspect of the general quality of life. We expanded upon the results of previous trials and provided data concerning the longevity and the precise effect of multiple sessions, bifrontal DLPFC tDCS. Our sample size (*n* = 39) was limited, which might have contributed to the lesser statistical power of the analyzed items.

**Clinical trial registration:**

[www.ClinicalTrials.gov], identifier [NCT05437185].

## Introduction

Tinnitus is the phantom perception of meaningless sounds in the absence of external noise. It is estimated to affect up to 15% of the population and about 1–2% in severe forms (1). It causes severe mental health issues (such as depression and anxiety), leading to disability and potentially suicide (2). There is no single etiological cause of tinnitus, more likely a plethora of different ones with variable manifestations (ringing, buzzing, cricket-like, hissing, whistling, or humming) (3, 4). Apart from a few specialized centers dedicated to tinnitus research, the care is usually dispersed between various neurological, otorhinolaryngological, psychiatric, rehabilitation, and general practitioner departments using different treatment approaches from the fields of psychotherapy (5), physical therapy (6), pharmacology (7), neurostimulation (8), and even surgery (9, 10). The research in the field of non-invasive brain stimulation has provided some potent therapeutical approaches, such as repetitive transcranial stimulation therapy (11) or transcranial direct current stimulation (12). Tinnitus is often accompanied by the symptoms of depression (in up to 33% of cases) (13) and anxiety (in up to 32% of patients) (14), and their relationship with tinnitus seems to be bidirectional. In the case of depression, tinnitus is usually thought to aggravate depressive symptoms and vice versa (15). Interestingly, neuronal circuits activated in both the patients with depression and tinnitus have been discovered, as well as similar changes in the hypothalamic–pituitary–adrenal axis, which might argue against pure comorbidity by coincidence (15). Anxiety is hypothesized to play a role in the cognitive model of tinnitus, which emphasizes the incorrect interpretations of the symptoms and the negative reinforcement of the distress (16, 17). Similarly, some overlaps in certain neural networks and ambiguous involvement of the hypothalamic-pituitary–adrenal axis have been proposed in the relationship between anxiety and tinnitus (18). It is also thought that proper screening and therapeutic approaches to anxiety could improve the perceived tinnitus level (18). Considering the profound impact on mental health and quality of life, the investigation of potential treatment interventions is vital.

Transcranial Direct Current Stimulation (tDCS) is a popular brain stimulation method that utilizes the properties of weak direct current flowing through the neural tissue to elicit subthreshold action potential changes (19). The effect is complex and includes changes in a wide area of neurotransmitter systems, including dopaminergic (20, 21), serotoninergic (22), cholinergic (23), and glutamatergic (24). It affects the functional connectivity, plasticity, and synchronization within several brain networks and topological organizations (25–27), while not being limited only to neurons but also glial (28) and non-neuronal cells sensitive to electric fields (29). One of its main paradigms used to rely on the excitatory effect under anode while manifesting an inhibitory effect under cathode (30). Nowadays, this division does not seem as linear as many used to hypothesize (31). Furthermore, the excitatory or inhibitory effects might depend more on balance between the biochemical changes induced by the flow of the electric current (32, 33). Almost two decades of clinical trials have confirmed the safety profile, and no consistent evidence of life-threatening adverse effects exists (12). While tDCS provides a relatively safe and easy-to-use method of neurostimulation even for a layperson (34), it is currently not supported for unsupervised medical use en masse (35). The in-home use of tDCS requires careful considerations and competencies on the side of the health care provider, supervisor, patient, and tDCS device (36). All in all, the in-home use of tDCS has been on the rise (37), and the use of tDCS as a telemedicine-assisted method has increased in importance (38), mainly considering the rising need for telemedicine in COVID and post-COVID times (39). The use of tDCS in the treatment of tinnitus has been a subject of extensive research efforts since 2006 (30), and it is hypothesized that the associated affective symptoms (40) (if not tinnitus itself) (41) could be influenced by the use of a proper protocol (42). Even though short-term tinnitus suppression has been achieved (43), the long-term effect has not been thoroughly assessed yet (44). The most promising stimulation areas seem to be over the left temporoparietal area (LTA) and dorsolateral prefrontal cortex (DLPFC). In theory, the stimulation over LTA suppresses the loudness; the stimulation over DLPFC suppresses the annoyance of tinnitus (43). Even though some authors claim DLPFC to be involved in both loudness and annoyance suppression (40). DLPFC has been associated with several facilitatory and modulatory functions related to auditory memory storage, auditory attention, and input to the primary auditory cortex (45–48). Many tDCS protocols employed in tinnitus research differ by the stimulation location (LTA vs. DLPFC), electrical current (usually 1.5–2 mA), the number of sessions (usually 1–10), washout periods (less than 24 h and up to 96 h), and several other parameters, which might dilute a concentrated research effort to single out the most effective approach (12). All in all, tDCS might represent a promising therapeutic option, but further systematic research is warranted, especially regarding the long-term outcomes.

Our goal was to evaluate the effect of bifrontal tDCS with electrodes over both DLPFCs on tinnitus and associated anxiety, depression, and quality of life compared to sham (placebo). The study protocol employed in the trial was heavily inspired by the works of Shekhawat and Vanneste in 2017 (49) and 2018 (48) and Faber et al. in 2012 (40). Shekhawat et al. tried to optimize the protocol by comparing different current and stimulation parameters. They concluded the optimal approach to be anode over right DLPFC, cathode over left DLPFC, with no significant differences between 1.5 and 2 mA, between 20 and 30 min of stimulation, and between 6 and 10 sessions (48). Unfortunately, their trial lacked a placebo arm and a long-term follow-up. We decided to shorten the washout period and apply the six sessions in 2 weeks (compared to Shekhawat’s six sessions in 3 weeks) due to operational reasons. We decided to use the current of 1.5 mA compared to 2 mA due to possible higher safety for practical application (even though that might be negligible) (50). A 5 × 5 cm (25 cm^2^) electrodes (compared to Shekhawat’s 35 cm^2^) were utilized due to our limited access to bigger electrodes at the time. Given Shekhawat’s call for additional research using sham group and long-term effect assessment, we wanted to provide data missing in previous trials focusing on the extent of the effect and its longevity.

## Materials and methods

A prospective, randomized, double-blinded, placebo-controlled, two-arm trial was conducted at the Department of Psychiatry, General University Hospital in Prague, Czechia. The research was conducted in accordance with the Declaration of Helsinki and was approved by the Ethics Committee of General University Hospital in Prague, Czechia, in 2019 under the reference number 531/19 S-IV. The trial took place between September 2019 and February 2022.

### Recruitment

Participants were recruited through the recruitment campaign of the Department of Psychiatry, which was supported by outpatient services of several neurological, psychiatric, internal, and otorhinolaryngological departments in several university hospitals around Prague. All participants were required to sign written consent with the trial, anonymized data, the European Union General Data Protection Regulation (GDPR), and were fully informed about the trial’s goals, risks, and requirements. Participation was not associated with any financial reward.

### Inclusion and exclusion criteria

The participation was offered to persons at least 18 years of age (on the day of signing the consent) with a history of tinnitus lasting at least 6 months. We excluded persons contraindicated to tDCS – such as those with epilepsy, intracranial masses or metallic objects, pregnancy, and heart conditions. We also excluded persons with a history of alcohol and drug abuse, persons unwilling to sign the informed consent or persons who underwent any other tinnitus therapy in the last 6 months. We intended to discontinue the treatment in any participant developing any severe adverse effect (significant exacerbation of tinnitus, epileptic seizure, severe headache, or any adverse effect deemed severe enough by the participants) and in participants non-compliant or unwilling to participate further with the trial and its follow-ups. We did not require the participants to discontinue any medication. If any medication was used, we required a stable regimen for at least 6 months prior to the stimulation and during the follow-up.

### Sample

Based on our initial power analysis, 36 participants equally distributed between the groups should have provided us with the desired power of 95% with regards to an estimated improvement of 25% from a baseline of 50 (SD = 10). Clincalc.com Sample Size Calculator was used for the initial calculation (51).

In total, eighty-one persons were offered participation in the trial. Three of them refused to sign the informed consent and thus were encouraged to pursue other treatment options with their outpatient specialists. Thirty-four persons fulfilled at least one exclusion criterion and were not allowed to participate. Interestingly, the preliminary CT screening contributed to the early diagnosis and proper treatment of a brain tumor. One participant decided to withdraw the consent after two sessions of tDCS, and their data was deleted as required by the law. Four participants were non-compliant with the follow-up. As a result, 39 participants with non-pulsatile tinnitus underwent the stimulation and completed a series of follow-ups.

**FIGURE 1:**
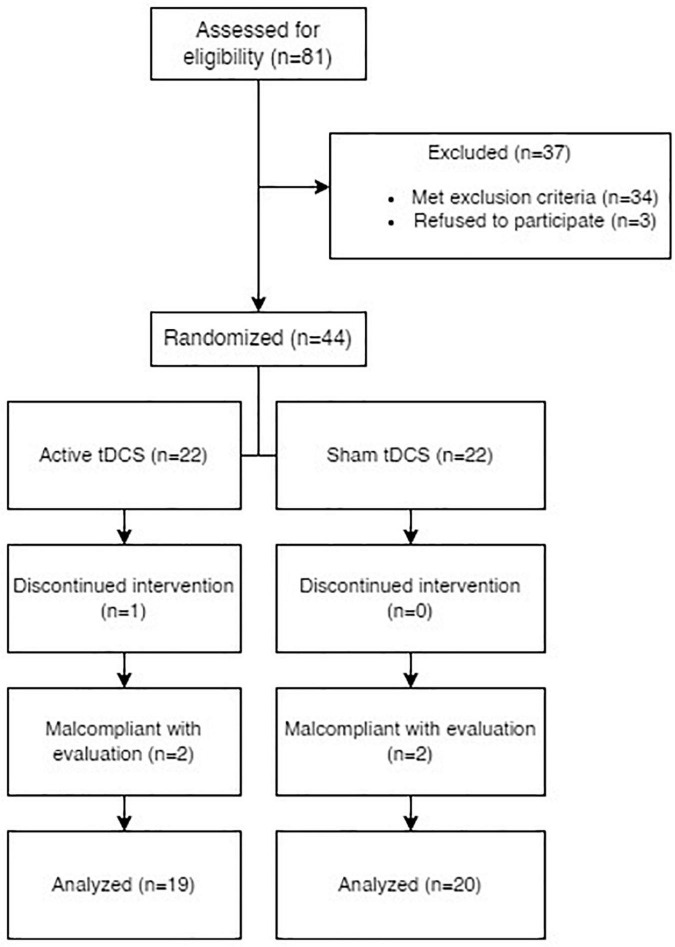
PRISMA diagram of the enrollment process and further compliance with follow-ups.

Our active group was comprised of 19 participants, while the sham group was comprised of 20 (See Table 1 for demographical data). Apart from symptoms of anxiety and depression, no participant had a history of schizophrenia or any type of psychotic disorder, somatoform disorder, or obsessive-compulsive disorder. All participants had a history of at least one previous therapeutic approach for tinnitus in the past and no stimulation treatment at least 6 months prior, concurrently, or 6 months after the tDCS (See Figure 1 for the PRISMA diagram).

**TABLE 1:** Demographic data of the participants with respect to the research arm (SD = standard deviation).

	Active group	Sham group
Females n (%)	8 (42.11%)	8 (40%)
Males n (%)	11 (57.89%)	12 (60%)
Non-binary n (%)	0 (0%)	0 (0%)
Average age	49 years (median = 50, SD = 16.73)	46.15 years (median = 42, SD = 18.5)
Average tinnitus duration	48.47 months (median = 36, SD = 57.6)	33.2 months (median = 23, SD = 33.42)

### Stimulation protocol and randomization

**FIGURE 2:**
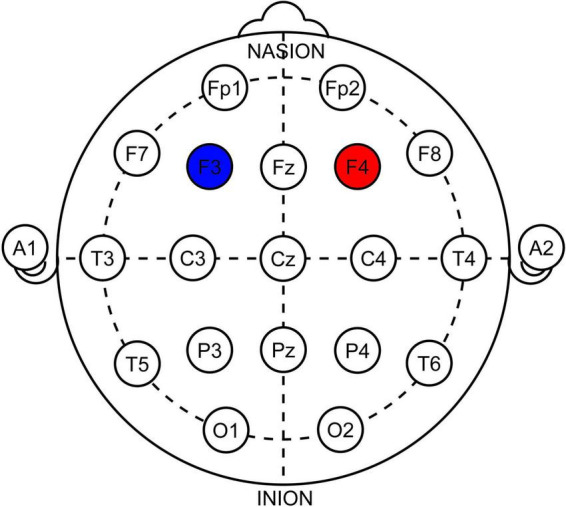
Illustration of electrode positioning in 10–20 system. Red-anode, Blue-cathode. Note that the electrodes were squares 5 × 5 cm, not rounds as depicted.

We aimed for six sessions of anodal stimulation over the right DLPFC (F4 in 10–20 EEG system) with cathode above the left DLPFC (F3) using HDCstim by Newronika S.r.l., Italy. The therapy was administered over 2 weeks (Mon, Wed, Fri) to ensure a washout period of 48 to 72 h between applications. The current of 1.5 mA was delivered via silicone electrodes inserted into saline (0.9%) filled cellulose sponges, both 5 × 5 cm (Current Density of 0.6 A/m^2^), for 20 min with 20 s of both ramp-up and ramp-down. The sham (placebo) was administered using the same devices with a preprogrammed sham protocol (using HDCprog by Newronika S.r.l., Italy) of 20 min to be virtually indistinguishable from the active stimulation. An International 10–20 EEG system was used to determine the stimulation location, and dedicated EEG caps were used to ensure consistency between applications (See Figure 2).

The participants were distributed between 2 arms using an aperiodic, non-deterministic, atmospheric random noise randomization algorithm (52). The stimulation type remained blinded until the completion of the last follow-up (6 months after the last stimulation); thus, all the data had been gathered from properly blinded participants. Earlier unblinding was done in participants who decided to drop out either by their own decision or by non-compliance with follow-ups. Data gathered (if any) from these participants were excluded per the initial design and were not statistically analyzed. The blinding was ensured by a dedicated team member responsible for programming the tDCS devices with no direct access to the participants or their data.

### Assessment

We aimed for four major assessment points – T1 before the stimulation, T2 immediately after the final (6th) stimulation session, T3 as a follow-up 6 weeks after T2, and T4 6 months after T2.

#### Symptoms of tinnitus

Tinnitus Functional Index (TFI) (53) was used as the main evaluating questionnaire. TFI is a questionnaire developed to evaluate tinnitus severity and negative impact. It employs 25 items (rated 0–10 or 0–100 by the increments of 10) to calculate the overall score on a scale of 0–100 (higher generally means more severe). It also calculates eight subdomains -TFI Intrusive, TFI Sense of Control, TFI Cognitive, TFI Sleep, TFI Auditory, TFI Relaxation, TFI Quality of Life, and TFI Emotional – each corresponding to a subdomain impaired by tinnitus (each subdomain score calculated on a scale 0–100 – a higher number denotes a higher severity). Examples of TFI items include “How STRONG or LOUD was your tinnitus over the past week?”, “Did you feel IN CONTROL in regard to your tinnitus over the past week?” or “Over the PAST WEEK, how much has your tinnitus interfered with your ENJOYMENT OF LIFE?”. TFI has been shown to indicate a high internal consistency and reliability. Its limits lie in the unequal contribution of its subdomains to the overall tinnitus severity (54).

Iowa Tinnitus Handicap Questionnaire version 1 (ITHQ) (55) was used to supplement TFI. ITHQ is a 27-item questionnaire (each rated on a scale of 0–100) and calculates an overall score and three subdomains (factors) of tinnitus (each on a scale of 0–100 as well – a higher number denotes higher severity). Factor 1 is related to the social, emotional, and behavioral effects of tinnitus, Factor 2 is related to tinnitus and hearing, and Factor 3 focuses on the tinnitus outlook. Examples of ITHQ items include “I do not enjoy life because of tinnitus”, “Tinnitus makes me feel annoyed” or “I complain more because of tinnitus.” The questionnaire has been shown to manifest high internal consistency overall and in Factors 1 and 2, but not in Factor 3 (55). Moreover, its focus is slightly shifted toward the emotional aspects of the disease since about half of the items are related to them (56).

#### Symptoms of anxiety and depression

The symptoms of anxiety and depression were assessed using Beck Anxiety Inventory (BAI) (57) and Zung Self-Rating Depression Scale (SDS) (58). BAI is a standardized and frequently used self-evaluating method. The frequency of 21 signs of anxiety (i.e., “feeling hot,” “handshaking,” or “fear of dying”) is evaluated for their frequency on a scale of 0–3 (a higher number denotes a higher frequency). The severity of the reported anxiety is based upon the overall value. Its limitations include the subjective nature of the questionnaire and its focus on the physical signs of anxiety (more congruent with panic attacks). SDS is also a standardized and popular self-evaluating method used to evaluate 20 items related to depression (each on a scale of 1–4 with several items rated negatively). The raw overall score is converted to the SDS index, which indicates the severity of depressive symptoms. Examples of its items include “I feel down hearted and blue,” “I notice that I am losing weight” or “I find it easy to make decisions.” Its limitation is the subjective nature compared to clinician-rated tools such as the Montgomery–Åsberg Depression Rating Scale or the Hamilton Depression Rating Scale.

#### The general quality of life

WHO-Quality of Life-BREF (59) was used to evaluate changes in the perceived quality of life during the trial. It is an instrument developed by the WHO to cover a broad spectrum of items in the quality of life. The questionnaire defines the quality of life as the position of an individual in the context of their culture, life goals, expectations, lifestyle a hobby. The brief version contains 26 items and calculated four domains of the quality of life – domain 1 (physical domain), domain 2 (psychological domain), domain 3 (social relations), and domain 4 (environment). Each domain is calculated based on the rated items, and their raw scores are converted into a scale of 0–100 (a higher number denotes a higher quality of life). Examples of its items include “How would you rate your quality of life?”, “How much do you enjoy life?” or “How satisfied are you with yourself?”. WHO-QoL-BREF has shown adequate internal consistency and is considered a sound instrument, even though its domains were found to be strongly intertwined in some populations (60).

High internal consistency reliability of the questionnaires above was confirmed by our calculations as well (See Table 2 for Cronbach’s alpha).

**TABLE 2:** Internal consistency reliability of the questionnaires used expressed as Cronbach’s alpha at each point of evaluation.

	T1	T2	T3	T4
TFI	0.9707	0.9642	0.9702	0.9725
ITHQ	0.9285	0.9369	0.9257	0.9441
BAI	0.9013	0.904	0.9274	0.9154
SDS	0.9046	0.9083	0.9157	0.9135
WHO-QoL	0.9157	0.936	0.9327	0.9419

TFI, Tinnitus Functional Index; ITHQ, Iowa Tinnitus Handicap Questionnaire; BAI, Beck Anxiety Inventory; SDS, Zung Self-rating Depression Scale; WHO-QoL, World Health Organization Quality of Life abbreviated version.

**TABLE 3:** Assessment points and methods included in them.

Designation	Weeks since T1	Assessment
T1	0	TFI, ITHQ, BAI, SDS, WHO-QoL-BREF
T2	2	TFI, ITHQ, BAI, SDS, WHO-QoL-BREF, blinding
T3	8	TFI, ITHQ, BAI, SDS, WHO-QoL-BREF
T4	24	TFI, ITHQ, BAI, SDS, WHO-QoL-BREF

Six sessions of tDCS were applied in 2 weeks between T1 and T2. Adverse effects were assessed between T1 and T2 during each session. TFI, Tinnitus Functional Index; ITHQ, Iowa Tinnitus Handicap Questionnaire; BAI, Beck Anxiety Inventory; SDS, Zung Self-rating Depression Scale; WHO-QoL, World Health Organization Quality of Life abbreviated version.

#### Blinding and adverse effects

Adverse effects were assessed in every participant during each session. Each participant was encouraged to guess what their stimulation type was supposed to be (between the choices of “active,” “sham,” or “do not know”). Blinding was assessed after the final stimulation in each participant, and the blinding index was calculated (61) (See Table 3 for the assessment points and evaluation methods included).

### Statistical analysis

Differences in outcomes in T2, T3, and T4 relative to T1 (i.e., delta T2-T1, delta T3-T1, and delta T4-T1) were compared between the intervention and sham groups using a non-parametric Mann-Whitney *U*-test. *P*-values were further adjusted for multiple comparisons by a Holm method. Adjusted *p*-values less than 5% were considered statistically significant. Analysis was performed in R statistical package, version 3.6.3 (62). Only participants who underwent six stimulation sessions and completed all evaluation points were included in the analysis. No incomplete data were included in the analysis; participants with missing data (non-compliant) were excluded per the original research protocol. *Post hoc* power analysis was based on Monte Carlo simulations with 10.000 repetitions.

## Results

### Symptoms of tinnitus

The median score in TFI of the active group at T1 was 58.4. The median score of subdomains was calculated as follows: TFI Intrusive 80, TFI Sense of control 56.67, TFI Cognitive 60, TFI Sleep 50, TFI Auditory 40, TFI Relaxation 50, TFI Quality of life 45 and TFI Emotional 56.67.

Compared to the TFI of the sham group at T1, which was 41.2. The median score of subdomains was calculated as follows: TFI Intrusive 56.67, TFI Sense of control 51.67, TFI Cognitive 33.33, TFI Sleep 36.67, TFI Auditory 30, TFI Relaxation 48.33, TFI Quality of life 35, and TFI Emotional 43.33 (See Table 4 for average, median, and SD values).

**TABLE 4:** Summary of the baseline averages, medians and SDs of all questionnaires and their subdomains at T1 in both active and sham groups.

Stimulation type	Average	Median	SD	Average	Median	SD	U statistic	*P*-value
			
	Active stimulation at T1			Sham stimulation at T1			Comparison between groups at T1	
TFI Total	53.890	58.400	23.290	45.520	41.200	18.910	144.500	0.206
TFI-Intrusive	69.470	80.000	26.630	60.500	56.670	18.930	142.000	0.181
TFI-Sense of Contol	57.540	56.670	23.830	49.330	51.670	14.730	163.500	0.464
TFI-Cognitive	51.580	60.000	26.420	38.830	33.330	21.830	130.500	0.096
TFI-Sleep	51.490	50.000	28.870	46.330	36.670	30.610	174.000	0.662
TFI-Auditory	44.390	40.000	31.370	32.170	30.000	25.550	151.000	0.278
TFI-Relaxation	56.670	50.000	26.670	50.670	48.330	27.090	161.500	0.431
TFI-Quality of Life	46.320	45.000	27.620	37.500	35.000	22.970	154.000	0.318
TFI-Emotional	56.320	56.670	29.560	51.500	43.330	25.830	165.000	0.491
ITHQ-Total	52.850	64.440	22.970	54.620	55.740	15.190	200.500	0.780
ITHQ-SEB	58.960	66.670	26.050	61.220	63.330	16.330	186.000	0.922
ITHQ-TH	41.590	37.500	28.060	44.000	43.130	23.520	209.500	0.593
ITHQ-O	52.500	52.750	18.090	51.130	50.000	14.990	179.500	0.778
Beck Anxiety Inventory	9.840	7.000	8.430	13.500	12.500	9.570	238.500	0.176
Zung-D-SDS index	53.890	55.000	12.080	49.550	44.000	15.620	151.500	0.285
WHO-QoL-Domain1	57.710	57.140	20.390	52.040	55.360	14.670	155.500	0.338
WHO-QoL-Domain2	61.840	62.500	16.150	52.080	42.170	16.860	131.000	0.099
WHO-QoL-Domain3	62.720	66.670	18.290	50.830	50.000	17.500	124.000	0.063
WHO-QoL-Domain4	64.310	65.630	12.810	63.130	65.630	11.130	176.000	0.703

Comparison of baseline values between groups at T1 included. TFI, Tinnitus Functional Index; ITHQ, Iowa Tinnitus Handicap Questionnaire; BAI, Beck Anxiety Inventory; SDS, Zung Self-rating Depression Scale; WHO-QoL, World Health Organization Quality of Life abbreviated version; SD, standard deviation.

**FIGURE 3:**
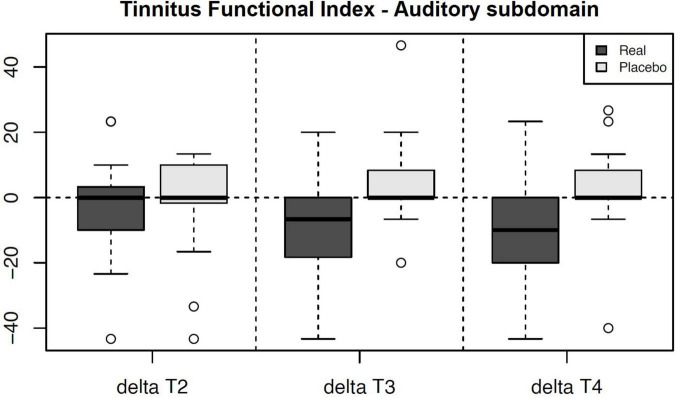
Chart depicting the outcome changes in the Auditory subdomain of Tinnitus Functional Index compared to the T1 baseline and their side-by-side comparison between the arms. The results in T3 and T4 are statistically significant a denote a decrease (improvement) in the Auditory subdomain of the active group. Note – Real = Active Group, Placebo = Sham Group.

**FIGURE 4:**
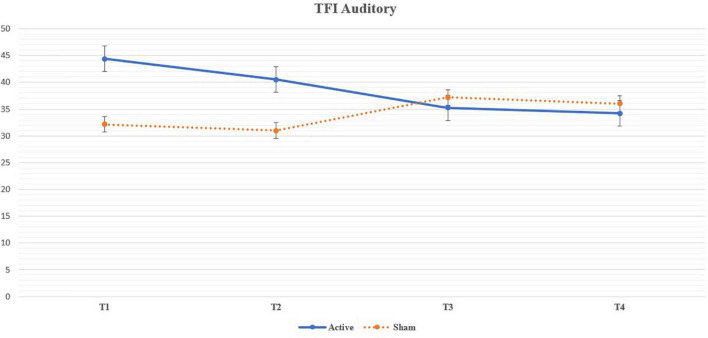
Line chart depicting the mean values of the Auditory subdomain of Tinnitus Functional Index throughout the trial and their side-by-side comparison between the arms. The results in T3 and T4 are statistically significant a denote a decrease (improvement) in the Auditory subdomain of the active group.

We noticed a statistically significant improvement in the Auditory subdomain of TFI in T3 (*U* = 87.5, *p* = 0.004; adjusted *p* = 0.035) and T4 (*U* = 92, *p* = 0.005; adjusted *p* = 0.049) compared to the sham group. The mean improvement in the active group at T3 (compared to T1) was 9.13 (median 6.7, SD 15.82) and at T4 (compared to T1) 10.17 (median 10, SD = 16.57) (See Table 5 for changes between the evaluation points in TFI Auditory, see Figure 3 for their comparison, and see Figure 4 for the side-by-side comparison of both groups).

**TABLE 5:** Changes in the TFI Auditory subdomain and their comparison to T1 (baseline) evaluation.

	Minimal	1st Quartile	Median	Mean	3rd Quartile	Maximal	SD
delta T2 Active	−43.3	−10	0	−3.8684	3.3	23.3	15.313
delta T2 Sham	−43.3	−0.825	0	−1.16	10	13.4	14.961
delta T3 Active	−43.3	−18.35	−6.7	−9.1263	0	20	15.821
delta T3 Sham	−20	0	0	5	7.525	46.6	13.302
delta T4 Active	−43.3	−20	−10	−10.174	0	23.3	16.568
delta T4 Sham	−40	0	0	3.835	7.525	43.3	15.937

The differences of active versus placebo were significant at T3 and T4. TFI, Tinnitus Functional Index; SD, standard deviation.

Noticeable trends were shown by the subdomains of Relaxation in T3 (*U* = 97, *p* = 0.009, adjusted *p* = 0.057) and T4 (*U* = 109, *p* = 0.021, adjusted *p* = 0.172), Quality of life in T3 (*U* = 96, *p* = 0.008, adjusted *p* = 0.057), Intrusiveness in T3 (*U* = 93.5, *p* = 0.006, adjusted *p* = 0.051) and by the Total score of TFI in T3 (*U* = 110, *p* = 0.025, adjusted *p* = 0.127). However, these results did not survive the adjustment of the *p*-values for multiple comparisons.

The median score in ITHQ of the active group at T1 was 64.44. The median scores of its subdomains were calculated as follows: ITHQ Factor 1 (Social, emotional, and behavioral) 66.67, ITHQ Factor 2 (Tinnitus and hearing) 37.5, and ITHQ Factor 3 (Outlook) 52.75.

Compared to the ITHQ of the sham group at T1, which was 55.74. Its subdomains were calculated as follows: ITHQ Factor 1 (Social, emotional, and behavioral) 63.33, ITHQ Factor 2 (Tinnitus and hearing) 43.13, and ITHQ Factor 3 (Outlook) 50 (See Table 4 for median and SD values).

The results in ITHQ and its subdomains did not reach statistical significance after *p*-value adjustments for multiple comparisons. Specific trends were noticed in Total ITHQ in T3 (*U* = 115, *p* = 0.035, adjusted *p* = 0.106) and its Tinnitus and hearing subdomain in T3 (*U* = 110.5, *p* = 0.026, adjusted *p* = 0.104).

### Symptoms of anxiety and depression

Initial evaluation of the active group showed 10 participants (54.63%) with no signs of anxiety, 5 participants (26.32%) with mild anxiety, 2 participants (10.53%) with moderate anxiety, and 2 participants (10.53%) with severe anxiety according to BAI. In comparison, the initial evaluation of the sham group showed 7 participants (35%) with no signs of anxiety, 6 participants (30%) with mild anxiety, 4 participants (20%) with moderate anxiety, and 2 participants (15%) with severe anxiety. The median score in BAI of the active group at T1 was 7 compared to 12.5 of the sham group. There was no statistically significant improvement in BAI (See Table 4 for average, median and SD values; see Table 6 for test statistic and *p*-values).

**TABLE 6:** Statistical significance of our outcomes at various evaluation points measured as a difference relative to T1 using a non-parametric Mann-Whitney *U*-test and further adjusted for multiple comparisons by the Holm method.

	T2 *P*-value	T2 adjusted	U statistic	T3 *P*-value	T3 adjusted	U statistic	T4 *P*-value	T4 adjusted	U statistic
TFI Total	0.298	1.000	152.5	0.025	0.127	110.0	0.063	0.444	123.5
TFI-Intrusive	0.744	1.000	178.0	0.006	0.051	93.5	0.080	0.482	128.0
TFI-Sense of Contol	0.270	1.000	150.5	0.276	0.735	151.0	0.283	1.000	152.0
TFI-Cognitive	0.348	1.000	156.5	0.145	0.580	138.0	0.476	1.000	164.5
TFI-Sleep	0.592	1.000	209.0	0.989	0.989	189.0	0.348	1.000	157.5
TFI-Auditory	0.311	1.000	154.0	0.004	**0.035**	87.5	0.005	**0.049**	92.0
TFI-Relaxation	0.298	1.000	153.0	0.009	0.057	97.0	0.021	0.172	109.0
TFI-Quality of Life	0.516	1.000	166.5	0.008	0.057	96.0	0.100	0.500	131.5
TFI-Emotional	0.886	1.000	184.5	0.245	0.735	148.5	0.490	1.000	165.5
ITHQ-Total	0.292	1.000	152.0	0.035	0.106	115.0	0.064	0.255	123.5
ITHQ Factor 1	0.481	1.000	154.5	0.122	0.244	134.5	0.066	0.255	124.0
ITHQ Factor 2	0.832	1.000	182.0	0.026	0.104	110.5	0.071	0.255	125.5
ITHQ Factor 3	0.646	1.000	206.5	0.240	0.244	148.5	0.897	0.897	195.0
BAI	0.035	0.104	265.0	0.453	0.453	217.0	0.442	0.442	217.5
SDS index	0.955	0.955	192.5	0.323	0.323	154.5	0.607	0.607	208.5
SDS item 20	0.955	1.000	190.0	0.323	0.645	209.5	0.607	1.000	180.5
WHO-QoL-BREF-Domain1	0.140	0.270	138.5	1.000	1.000	189.5	0.285	0.569	152.0
WHO-QoL-BREF-Domain2	0.006	**0.023**	96.0	0.090	0.361	133.5	0.050	0.198	124.5
WHO-QoL-BREF-Domain3	0.090	0.270	133.5	0.304	0.908	155.5	0.058	0.198	127.5
WHO-QoL-BREF-Domain4	0.113	0.270	134.0	0.303	0.908	155.0	0.989	0.989	189.0

Adjusted p-values considered significant are marked in bold. TFI, Tinnitus Functional Index; ITHQ, Iowa Tinnitus Handicap Questionnaire; BAI, Beck Anxiety Inventory; SDS, Zung Self-rating Depression Scale; WHO-QoL, World Health Organization Quality of Life abbreviated version.

According to SDS, the evaluation of the active group showed 7 participants (36.84%) with no depressive symptoms, 5 participants (26.32%) with signs of mild depression, 6 participants (31.58%) with moderate depressive symptoms, and 1 participant (5.26%) with severe depressive symptoms upon T1. The sham group contained 11 participants (55%) with no depressive symptoms, 3 participants (15%) with signs of mild depression, 3 participants (15%) with moderate depressive symptoms, and 3 participants (15%) with severe depressive symptoms. The median SDS index of the active group at T1 was 55 compared to the sham group of 44. The stimulation did not cause any significant changes (See Table 4 for average, median and SD values; see Table 6 for test statistic and *p*-values).

### The general quality of life

The initial (T1) median scores in WHO-QoL-BREF of the active group were 57.14 in Domain 1, 62.5 in Domain 2, 66.67 in Domain 3, and 65.63 in Domain 4. Compared to the median initial scores of the sham group, which were 55.36 in Domain 1, 42.17 in Domain 2, 50 in Domain 3, and 65.63 in Domain 4 (See Table 4 for average, median and SD values; see Table 6 for test statistic and *p*-values).

There was a statistically significant negative difference in Domain 2 at T2 (*U* = 96, *p* = 0.006; adjusted *p* = 0.023) compared to the sham. Other domains did not reach statistical significance. Upon noticing the change in WHO-QoL-BREF Domain 2 in T2, a comparison with SDS question 20 (I still enjoy the things I used to do) was made. Further analysis showed no significant change when comparing the differences in the outcomes of the SDS question 20 between the active and sham groups at each follow-up point. Furthermore, Spearman’s rank correlation coefficient came up without any significance.

Baseline (T1) comparison between active and sham groups showed no significant results in any questionnaires or their subdomains (See Table 4 for internal statistic and *p*-values). The complete list of changes in the questionnaires used during the trial is included as Supplementary Material. The *p*-values and test statistics of all statistical comparisons made are included in Table 6. *Post hoc* power analysis of all results regarding the employed sample size is included in Table 7.

**TABLE 7:** Statistical power of our results based on a *post hoc* power analysis.

	T2 ph power	T3 ph power	T4 ph power
TFI Total	0.075	0.749	0.434
TFI-Intrusive	0.118	0.615	0.408
TFI-Sense of Contol	0.267	0.365	0.234
TFI-Cognitive	0.074	0.447	0.319
TFI-Sleep	0.058	0.088	0.123
TFI-Auditory	0.074	0.809	0.714
TFI-Relaxation	0.048	0.624	0.386
TFI-Quality of Life	0.042	0.741	0.140
TFI-Emotional	0.045	0.329	0.128
ITHQ-Total	0.202	0.092	0.137
ITHQ Factor 1	0.187	0.118	0.153
ITHQ Factor 2	0.258	0.289	0.163
ITHQ Factor 3	0.077	0.125	0.112
BAI	0.333	0.142	0.150
SDS index	0.122	0.045	0.139
SDS item 20	0.052	0.061	0.059
WHO-QoL-BREF-Domain1	0.509	0.076	0.212
WHO-QoL-BREF-Domain2	0.678	0.468	0.523
WHO-QoL-BREF-Domain3	0.507	0.324	0.574
WHO-QoL-BREF-Domain4	0.563	0.250	0.077

Post hoc power analysis was based on Monte Carlo simulations with 10.000 repetitions. Ph, Post hoc; TFI, Tinnitus Functional Index; ITHQ, Iowa Tinnitus Handicap Questionnaire; BAI, Beck Anxiety Inventory; SDS, Zung Self-rating Depression Scale; WHO-QoL, World Health Organization Quality of Life abbreviated version.

### Blinding and adverse effects

James Blinding Index of the study was 0.8077 (95% CI 0.6931 – 0.9223). The upper bound of CI over 0.5 suggests that the blinding process was acceptable. Generally, more study participants were incorrect regarding their stimulation type rather than correct.

Throughout six sessions, adverse effects were reported 14 times – 7 (35%) participants in the sham group and 6 (31.58%) participants in the active group. Adverse effects were reported in 5.8% of sham and 5.3% of active sessions. Apart from itching, burning sensation, fatigue, tingling, and headache, one participant decided to discontinue the study after two active stimulation sessions claiming subjective exacerbation of tinnitus. The patient did not answer our further request to assess this phenomenon, asked for the removal of their data, and cut further contact (see Table 8).

**TABLE 8:** Adverse effects as reported in the trial.

Adverse effect	Sham (n)	Sham (%)	Sham (%) per all sessions	Active (n)	Active (%)	Active (%) per all sessions
Itching	2.00	10.00	1.67	2.00	10.53	1.75
Burning sensation	1.00	5.00	0.83	1.00	5.26	0.88
Fatigue	2.00	10.00	1.67	1.00	5.26	0.88
Tingling	1.00	5.00	0.83	1.00	5.26	0.88
Headache	1.00	5.00	0.83	1.00	5.26	0.88
Total	7.00	35.00	5.83	6.00	31.58	5.26

Each report was considered a separate case. The percentage taking the number of stimulations done was included for comparison. The participant claiming exacerbation but refusing further assessment was omitted.

## Discussion

Our clinical trial aspired to provide data regarding the extent and longevity of bifrontal tDCS (20 min, 1.5 mA, six sessions, anode over right DLPFC) with increased washout periods (48–72 h) in a prospective, randomized, double-blinded, sham-controlled setting on tinnitus, depression, anxiety and quality of life. As we mentioned, the stimulation protocol was heavily influenced by Shekhawat and Vanneste in 2017 (49) and 2018 (48) and Faber et al. in 2012 (40), and the aims were strongly shaped by their previous findings.

Based on our data, a delayed stimulation effect compared to Shekhawat’s immediate one was noticed. The difference between active and sham in some TFI subdomains started to manifest 6 weeks after the last tDCS session (T3) and was still noticeable at the final follow-up in 6 months (T4). The effect in TFI Auditory subdomain survived the adjustments for multiple comparisons and was statistically significant at T3 and even T4 (6 months later). We did not notice any significant immediate effect (T2) on tinnitus symptoms.

We hypothesize that the effect build-up might occur in the third week since the beginning of the stimulation because there was a difference in the timespan (our six sessions over 2 weeks versus Shekhawat’s six sessions over 3 weeks) (48). The immediate effect of their trial might also be explained by the placebo effect since they lacked a placebo arm. The delayed effect (63) of tDCS is an exciting phenomenon that possibly stems from the changes in neuroplasticity induced by the tDCS. Its clinical relevance is currently being assessed in the treatment of depression (64). We also noticed a similar effect pattern in our previous neuromodulatory research of repetitive transcranial magnetic stimulation in tinnitus, where changes in neuroplasticity had been proposed as well (65).

While Shekhawat et al. used a ten-point scale to evaluate loudness (48) and Faber et al. used a custom questionnaire based on the Visual Analog Scale (40), we used the TFI questionnaire as our primary tool (50), which might have provided us with a more precise tinnitus assessment. We noticed that the metrics of Shekhawat and Fabri might correspond more to the Intrusive subdomain of TFI, which showed a noticeable trend in T3. However, it did not survive further statistical analysis, which might have been caused by our limited sample size or the limits of previous studies. The same limitation might apply to TFI Relaxation in T3 and T4, TFI Quality of Life in T3, TFI Total score in T3, and Total ITHQ in T3, which initially showed a significant trend, but further adjustment of *p*-values decreased their statistical significance above the required level of 5%.

We theorize, and our current knowledge supports the theory that only certain domains of tinnitus are influenced by tDCS (40). There are also differences between the questionnaires. TFI evaluates eight subdomains compared to three subdomains of ITHQ. While a trend in tinnitus and hearing ITHQ subdomain in T3 was noticed, we acknowledge it does not entirely correspond to TFI auditory subdomain.

Our results seemingly show a slight initial increased level of anxiety in the sham group at T1, but no statistically significant baseline difference was present. We did not notice a significant effect on anxiety throughout the trial nor a significant change in the anxiety levels. Faber’s trial initially demonstrated a possible modulation of anxiety in tinnitus patients using a similar montage (anode over right-DLPFC, cathode over left-DLPFC) and an antidepressive effect using an opposite montage (assessed by Hospital Anxiety Depression Scale) (40). Their results did not survive a statistical comparison between active and sham groups.

The sample did not primarily focus on patients suffering from clinical depression, even though it frequently accompanies tinnitus (4). The reduction of tinnitus has already been demonstrated to lead to a reduction in depression (66). Our results suggest that the partial reduction in tinnitus has not been sufficient to influence the depressive symptoms significantly. The stimulation protocol has not been proven effective in the treatment of depression. Opposite montage with the anode over F3 and cathode over F4 is usually employed (67). All participants suffering from depressive syndrome (after completing the T4 evaluation) were offered rTMS treatment at our department and were encouraged to consult their general practitioner or outpatient psychiatrist.

The psychological domain (domain 2) of WHO-QoL-BREF encompasses positive feelings, thinking, esteem, body, negative feeling, and spirituality. The statistically significant negative difference in the perceived quality of life in T2 of the active group compared to the sham is not entirely clear to us. Considering a similar profile of adverse effects between the groups and no other significant difference in any questionnaires (or SDS question 20), including the blinding index, we theorize the active stimulation might have caused the participants a certain level of discomfort or negative feelings, which was not deemed worthy of reporting by the participants. However, we cannot be sure without any other reports of this phenomenon, and current literature has come up with no plausible explanation. Upon closer inspection of the data, a more pronounced positive effect in the sham group was noticed rather than the negative effect in the active group. That is either indicative of a placebo effect not seen in any other used metrics or an unexplained external, positive influence isolated on the psychological domain of the sham group. The isolated occurrence of this phenomenon was not reflected either in TFI Quality of life or SDS question 20. We did not notice the significance of this effect in further follow-up points, and it was shown to be transient.

On the contrary, we proved a significant positive effect on certain subdomains of tinnitus at T3 and T4, but the results were not reflected in any domain of WHO-QoL-BREF.

Retrospectively, a larger sample would enable us to detect lesser improvements with higher statistical power, even though our initial power analysis was performed with very rigorous levels of statistical power in mind. Moreover, we even surpassed our original plan of 36 participants. Baseline differences between the two groups on the measured variables came up without statistical significance. Thus, both groups were statistically comparable.

James Blinding Index showed a sufficient level of blinding above the required threshold (> 0.5), which suggests the participants not to be aware of (or rather mainly guessing incorrectly) their stimulation type in the trial.

The adverse effects are consistent with the current level of their understanding (61). One participant was excluded per their request due to claims of tinnitus exacerbation. Exacerbation is an adverse effect that supposedly results from the neuroplasticity changes during the stimulation (68). We found this phenomenon mostly in studies focusing on LTA stimulation, not DLPFC (68). Unfortunately, we could not confirm or assess the phenomenon since the participant requested the discontinuation of their participation and the removal of their data. We complied with their request, following the law.

## Conclusion

Compared to placebo, we reached a statistically significant long-term effect in reducing auditory difficulties associated with tinnitus lasting 6 months after six sessions of bifrontal tDCS. Moreover, we noticed several exciting, long-lasting trends in other subdomains of tinnitus, such as its intrusiveness, interference with relaxation, quality of life associated with tinnitus, and overall tinnitus scores. Interestingly, transient, short-term, negative change in the psychological aspect of quality of life was noticed.

The worldwide COVID pandemic might have contributed to a lesser willingness of potential patients to participate. We believe that the results might have been more convincing if a larger pool of participants had been used, which is the main limitation of our trial. Even with this limitation, we fulfilled our goal. We provided more precise data on the possible longevity and extent of bifrontal application of tDCS over multiple sessions with more extended washout periods. Thus, we were able to fill in these gaps in the previous trials. We also confirmed previous findings regarding the safety profile and tolerability of tDCS, even though exacerbations of tinnitus due to tDCS deserve further research.

## Data availability statement

The original contributions presented in this study are included in the article/Supplementary material, further inquiries can be directed to the corresponding author.

## Ethics statement

The studies involving human participants were reviewed and approved by the Ethics Committee of General University Hospital in Prague, Czechia. The patients/participants provided their written informed consent to participate in this study.

## Author contributions

TM: conceptualization, methodology, investigation, data curation, writing, project administration, and visualization. JA: verification and blinding. JBud, GP, TL, EM, KP, JH, and JBun: investigation. VC: formal analysis, software, data curation, and visualization. LK, JK, and VF: resources. MA: supervision, funding acquisition, and resources. All authors contributed to the article and approved the submitted version.
